# Editorial: Evidence and empathy: nursing at the crossroads of art and science

**DOI:** 10.1177/17449871251380130

**Published:** 2025-09-30

**Authors:** Sarah Bekaert

**Affiliations:** Faculty of Humanities and Social Sciences, Oxford Brookes University, Oxford, UK

Nursing today stands at a crossroads: increasingly scientific, increasingly technological, and yet always rooted in the deeply human work of care. The breadth of scholarship in this edition demonstrates that nursing is not a single voice, but a chorus: spanning randomised controlled trials (RCTs), instrument validation, qualitative explorations and even interpretive poetry. Together, these contributions illuminate the discipline’s central commitments: to empower and advocate for patients, to adapt care to culture and context, and to reflect on what it means to care in the fullest sense.

## Clinical interventions and empowerment

The opening group of intervention studies, which range from a telenursing empowerment programme for patients with chronic obstructive pulmonary disease (COPD), to the use of cold needles to ease dialysis pain, to instructional modules reducing discomfort after stent removal – remind us that nursing interventions are often both simple and profound. Grounded in science yet attentive to lived experience, they draw on theories of self-care and self-efficacy but take shape in pragmatic acts as modest as cooling a needle. Here, the science of nursing meets its art: evidence translated into small changes that transform daily realities.

The RCT of a telenursing empowerment programme for COPD patients (Rajabi et al., 2025) exemplifies the nurse as both educator and advocate. Rooted in Bandura’s (1977) self-efficacy and Orem’s (2001) self-care deficit theory, it demonstrates empowerment not as abstraction but as a process of enabling confidence and capability through accessible technology. This is vital at a time when health systems are strained. Equally, the studies on cold needle use in dialysis cannulation (Ozen et al., 2025) and instructional modules for stent removal (Dawood et al., 2025) showcase nursing’s pragmatic creativity. Low-cost, low-risk and immediately applicable, they highlight nursing’s role in alleviating suffering through thoughtful detail. This echoes Nightingale’s (1860) conviction that seemingly small adjustments can deliver profound benefits. At a moment when healthcare discourse is dominated by high-tech solutions, these studies reaffirm that nursing innovation is rooted in attention to the human experience.

## Expanding roles and perspectives

From clinical interventions, this edition moves into questions of scope and responsibility. The perspective piece on nutrition by Francesca Tabacchi (2025), tellingly titled *Beyond the cherry on top*, encourages us to reconsider how nutrition is positioned in nursing practice. Nutrition is not an ‘extra’, but central to patient recovery and well-being. This resonates with Jean Watson’s (2008) theory of human caring, which situates health not only in disease management but also in holistic attention to the contextual conditions that sustain life. In repositioning nutrition as core nursing work, we confront a broader issue: how easily fundamental aspects of care can be marginalised in systems dominated by biomedical priorities.

The paper on cognitive load and task complexity among clinical nurses (Zhang et al., 2025) extends this conversation into the intellectual labour of nursing. Nurses are not simply task performers, but they are knowledgeable workers navigating complex issues, prioritising under pressure and exercising judgement in real time. Exploring how cognitive load mediates guideline adherence, this study highlights the systemic challenges nurses face when demands exceed resources. The findings echo Patricia Benner’s (1984) novice-to-expert model, which illustrates how expertise is forged not merely through knowledge, but through the ability to manage complexity and nuance. In a world facing widespread nursing shortages and burnout, attention to cognitive burden is urgent.

## Qualitative insights into patient experience and meaning

If the first half of this issue emphasises what nurses do, the next reminds us to listen carefully to those who receive nursing care. The qualitative study of breast cancer survivors (Almallah et al., 2025), framed through the Health Belief Model (Rosenstock 1974), explores the cues and barriers influencing treatment initiation. Here, the nurse emerges as a partner in navigating fear, social context, financial strain and belief systems. The findings affirm that decision-making in health is never purely clinical; it is a complex negotiation of personal meaning, risk perception and support.

The development of the MENTOR HUB stroke rehabilitation framework for younger women builds on this insight (Tarihoran et al., 2025). By synthesising survivor and professional perspectives, it highlights the unique challenges of gender, age and identity in recovery. Similarly, the narrative inquiry into men living with lymphoedema interrogates masculinity in the context of chronic illness (Cooper et al., 2025). Both papers exemplify Parse’s (1992) human becoming theory and Watson’s (2008) caring science, which state that health is lived uniquely and must be understood within the stories people tell about themselves. These contributions are reminders that nursing knowledge is not only measured in scales and measures, but also in meanings, metaphors, and lived realities. They also speak to broader social issues such as gender equity in healthcare.

## Implementation and measurement

Translating knowledge into practice requires structures, tools, and systems. The participatory nutrition project in aged care offers a powerful example of implementation science in nursing (Lea et al., 2025). By engaging staff, residents and families in co-designed interventions, Lea et al. (2025) acknowledged the complexity of aged care environments and the need for collaborative change. Nutrition champions and participatory approaches emerge as facilitators, showing how sustainable improvement depends on culture as much as on evidence.

The validation of the Watson Caritas Patient Instrument in Spain (Delgado-Galeano et al., 2025) and the cross-cultural adaptation of the Scale for the Image of Nursing Profession in Italy (Godino et al., 2025) further expand nursing’s methodological toolkit. Such tools for measurement are not stand-alone exercises; they give quantifiable form to the phenomena of caring and professional identity. That these instruments require cultural adaptation underscores nursing’s global reach and the need to respect contextual differences while maintaining theoretical coherence. Together, these papers also remind us that nursing theory, when operationalised through robust instruments, strengthens the profession’s ability to demonstrate impact and advocate for change.

## Professional development, education and future leadership

No profession can thrive without nurturing its future. The survey of nurse researchers in Karnataka reveals significant barriers to publication, from lack of time to insufficient technical support (Rajendran et al., 2025). These challenges, while specific in the paper, resonate globally. If nursing scholarship is to flourish, investment in mentorship, structured training and supportive infrastructures is essential. Publication is not merely an academic exercise, but it is the means by which nursing knowledge becomes visible, shapes policy and secures its place within the sciences – goals we strive to fulfil in the *Journal of Research in Nursing*.

Equally, the reflections of senior nursing students on management and leadership highlight both gaps and opportunities in nursing education (Ferreira Aydogdu et al., 2025). The authors’ call for stronger preparation in leadership mirrors international priorities: the International Council of Nurses (2023) emphasises the role of nurses as leaders, advocates and policy shapers. By paying heed to student voices, we are reminded that leadership development must begin early, embedded in curricula that prepare nurses not only to deliver care but also to transform systems.

## Closing reflections: nursing’s art and science

This issue concludes with an interpretive poetry piece, *Sorted*, which explores injectable medications at the end of life (Madden and Bowers, 2025). Poetry may seem distant from RCTs and validation studies. Yet its inclusion is deliberate, and welcome. Poetry, like nursing, seeks meaning in the face of vulnerability. It invites us to dwell not only on what we know but also on what we feel, fear, and hope. In end-of-life care especially, the nurse’s role is as much about presence and recognition of humanity as it is about technical competence.

This closing contribution brings us full circle. Nursing is both art and science, evidence and empathy, intervention and reflection. Across the papers in this edition, we see the discipline at work: generating rigorous evidence, developing culturally sensitive measures, implementing change in complex systems, listening to patients’ voices, nurturing future leaders and attending to the human condition in all its fragility and strength.
